# Views of the Public and Healthcare Professionals on Newborn Screening for Spinal Muscular Atrophy and the Potential for Detecting Adult-Onset Types in Patients-in-Waiting in Hong Kong

**DOI:** 10.7759/cureus.79190

**Published:** 2025-02-17

**Authors:** Chloe M Mak, Jacky Kwan Ho Lee, Jimmy Chi Lap Wong, Chun Yiu Law, Chun Hei Toby Chan, Chun Wing Yeung, Shirley Pang, Edgar Hau, Sophelia Hoi Shan Chan

**Affiliations:** 1 Department of Pathology, Newborn Screening Laboratory, Hong Kong Children's Hospital, Hong Kong, CHN; 2 Department of Medicine, Queen Mary Hospital, Hong Kong, CHN; 3 Department of Clinical Genetics, Hong Kong Children’s Hospital, Hong Kong, CHN; 4 Department of Pediatrics and Adolescent Medicine, The University of Hong Kong, Hong Kong, CHN

**Keywords:** adult-onset sma, gene therapy, newborn screening, parental attitude survey, smn1, smn2, spinal muscular atrophy

## Abstract

Background and aim: Spinal muscular atrophy (SMA) is an inherited neuromuscular disease. Although drugs are now available to treat patients with SMA, newborn screening (NBS) is essential for the early identification and treatment of affected babies. However, the attitudes towards NBS for SMA, particularly the adult-onset type, vary among different stakeholders. While supporters advocate for NBS due to early diagnosis and intervention, skeptics argue that the uncertainty about the severity and disease onset age can cause unnecessary parental and patient anxieties. This study aimed to explore the knowledge, attitudes, and perspectives of the general public and healthcare professionals regarding NBS for SMA in Hong Kong.

Methods: A prospective online quantitative questionnaire survey was conducted on the general public, healthcare professionals, and the SMA community aged 18 years or above between December 2022 and February 2023. Participants were asked 28 questions about their knowledge of SMA, views on implementing NBS, concerns about the possibility of detecting adult-onset SMA, and the time of treatment. Descriptive analysis by Microsoft Excel (Redmond, WA: Microsoft Corp.) was employed to determine the absolute frequencies and percentages of interviewees’ responses to each question of the questionnaire.

Results: We received a total of 223 responses, of which 165 (74%) were from the general public and 58 (26%) from healthcare professionals. The majority, 94.6%, were in favor of NBS for SMA, primarily due to the potential for immediate treatment for affected infants (90.5%, n=191). When considering the possibility that NBS results could indicate adult-onset SMA, 87.9% (n=196) expressed a desire to know their child's results and 80.7% (n=180) were comfortable with their parents being informed of their NBS findings.

Conclusions: Both the general public and healthcare professionals expressed support for the implementation of NBS for SMA in Hong Kong, believing that early diagnosis and intervention can improve the quality of life of SMA patients. Proper public health education is crucial to help individuals make informed decisions about NBS for SMA.

## Introduction

Spinal muscular atrophy (SMA) is one of the leading genetic causes of infant mortality, and until recently, it was considered incurable [1-3]. However, the development of new treatments and gene therapy has changed this outlook. The estimated incidence rate for SMA is around one in 10,000 live births. Type 1 SMA (SMA1) accounts for more than half of all cases with an incidence of approximately six in 1,00,000 while type 4 SMA (SMA4) being the rarest making up less than 5% of all cases [1,2]. To enable early diagnosis and prompt treatment, newborn screening (NBS) for SMA is now available as a routine program in many parts of the world, including most states in the United States, Canada, and Belgium [4-6]. However, attitudes towards NBS for SMA, particularly concerning the possibility of detecting adult-onset SMA through NBS, vary among stakeholders, including the general public and healthcare professionals with different educational and cultural backgrounds.

SMA is an autosomal recessive disorder caused by partial or complete deletion or small mutations of the survival motor neuron 1 (SMN1) gene located on chromosome 5q13.2 [7]. It is most commonly caused by homozygous deletion of SMN1 gene (around 95% of SMA patients, mostly exon 7 and 8 deletions), with 2-5% of patients having compound heterozygous mutations or intragenic mutation in SMN1 allele [8,9]. Loss of SMN1 gene causes motor neurons in the spinal cord and brainstem to produce insufficient SMN proteins [10]. Consequently, these neurons degenerate and break down, preventing muscles from receiving proper signals from the brain. This leads to progressive skeletal muscle atrophy and muscle weakness, causing difficulty in moving the head and neck, sitting, crawling, walking, raising arms, and even breathing and swallowing [11]. Patients with SMA have normal cognition.

The clinical phenotypes of pediatric-onset SMA can be divided into three pediatric-onset subtypes (type 1, 2, and 3) and an adult-onset subtype (type 4) based on disease severity and age of onset. The most severe form, SMA1 or "Werdnig-Hoffman disease," manifests during the first six months of life. Without respiratory assistance, children with SMA1 typically die within the first two years [12]. Type 2 SMA (SMA2) occurs between the ages of six and 18 months and is associated with a lower life expectancy than that of the general population [13]. Type 3 or Kugelberg-Welander disease (SMA3) develops symptoms after 18 months of age and has a life expectancy similar to the general population [14]. All three subtypes of pediatric-onset SMA experience progressive muscle weakness and loss of motor skills with age. In SMA4, also called adult-onset SMA, symptoms can occur after 30 years of age [15]. This is the rarest and mildest form of SMA, with mild-to-moderate muscle weakness, tremors, and mild breathing problems. Patients can remain mobile or asymptomatic with normal life expectancy.

Most individuals have two copies of SMN1 gene and one to two copies of SMN2 gene, with more frequent copy number variations in SMN2 [16]. While the onset of SMA is due to homozygous loss of SMN1, the disease subtypes are modulated by the copy numbers of SMN2. The functional difference between SMN1 and SMN2 is due to a nucleotide C-to-T transition in exon 7, which causes most SMN proteins produced by SMN2 to be unstable and not fully functional. Since both SMN1 and SMN2 encode the same SMN proteins, the remaining functional SMN proteins produced by SMN2 can compensate for some loss of SMN1 [17]. The number of SMN2 copies varies among individuals ranging from zero to eight [16]. Generally, the fewer SMN2 copies an individual has, the more severe their phenotype is [16]. A hybrid SMN gene, formed by gene conversion from SMN1 to SMN2, has been discovered [8,18]. In terms of its correlation with clinical phenotype, it has been observed that the hybrid SMN gene appears to be related to milder forms of SMA [18].

Since 2016, treatment for SMA has become available for patients, with the following three ultra-expensive FDA-approved drugs for SMA: nusinersen, risdiplam, and onasemnogene abeparvovec. Treatment is indicated in all types of SMA in Japan and USA. Nusinersen and risdiplam are available to pediatric patients with SMA in Hong Kong supported by the Government via the Community Care Fund. Since 2018, all affected patients with SMA1 under the care of hospital authority who met the Nusinersen Expanded Access Program (EAP) inclusion criteria have been eligible for nusinersen treatment. Since 2019, in addition to SMA1 patients, pediatric patients with SMA2 and SMA3 have also been considered for treatment. All patients receive treatment during the symptomatic stage. The treatment progress is monitored by the SMA expert panel under hospital authority. At the time of writing, the third medication onasemnogene abeparvovec is under discussion for approval.

Evidence shows that administering treatment to asymptomatic newborns yields the best motor outcome. In the case of nusinersen, the Phase 2 NURTURE study (a five-year treatment period followed by a post-treatment follow-up evaluation) during the pre-symptomatic stage of SMA showed that after receiving the drug, all infants (n=25) in this study were alive without permanent ventilation and achieved independent sitting. Most of the studied infants (88%) were able to walk alone [19]. Two sham-controlled Phase 3 trials of nusinersen in children with SMA2 and SMA3 revealed that the majority of the affected children receiving the drug had a motor-milestone response and high likelihood of event-free survival [20,21].

When early treatment is necessary, NBS becomes a crucial next step. NBS can screen for newborns who may develop SMA at any onset period. Although a one-tier test detecting SMN1 exon 7 deletion is possible, the ideal NBS for SMA should provide genetic information on both SMN1 and SMN2 copy numbers. The detection of homozygous deletion of SMN1 exon 7 is widely adopted worldwide for NBS. However, not every laboratory conducts a supplementary test to provide timely information on SMN2 copy numbers for positive cases [22]. There are several challenges associated with the screening test. False-positive results may arise due to SMN1 hybrid genes or allelic dropout caused by the presence of interfering sequence variants in the SMN1 primer or probe binding sites. False-negative results may occur due to point mutations in the SMN1 gene [23,24]. Determining the copy number variation of the SMN2 gene can be achieved through methods, such as multiplex ligation-dependent probe amplification (MLPA) or digital polymerase chain reaction (dPCR). The initial result requires confirmation from another independent laboratory, necessitating the standardization of laboratory procedure for determining SMN2 copy number [25]. Additionally, since early treatment is vital for severe SMA1 and SMA2 patients, the turnaround time ideally should not exceed five working days due to therapeutic relevance [26]. This poses a challenge to laboratories, especially during long consecutive holidays.

Currently, a pilot NBS for SMA has been implemented in public hospitals in Hong Kong since October 2023. Through the NBS SMA program, affected babies can be identified at the pre-symptomatic stage and receive the earliest possible SMA treatment. However, there are complex issues and the probability of screening SMA babies with four or more SMN2 copies. As mentioned above, number of SMN2 copies correlates with the phenotype of SMA patients. Still, it is difficult to predict the clinical onset of the disease, as patients may have childhood, adolescent, or adult-onset, or asymptomatic for life with more than or equal to four SMN2 copies [23]. Conversely, it is possible to have severe symptoms even if one has more than or equal to five copies of SMN2 gene [27]. Close monitoring is an approach for this issue, while there may be default follow-up due to psychological stress [25]. Concerning the course of treatment, it is uncertain whether treatment should be initiated for asymptomatic patients with four SMN2 copies, given the extremely high cost of treatment and the possibility that the individual may never develop symptoms. However, a certain percentage of such individuals could develop pediatric onset SMA. Currently, treatment for asymptomatic babies with four SMN2 copies is advocated in the United States and China [28,29].

The American Academy of Pediatrics (AAP) and American College of Medical Genetics and Genomics (ACMG) recommend all children to undergo NBS, after providing education and counseling on the procedure, benefits, risks, and management of positive results of NBS [30]. However, some might argue that the association between NBS for rare diseases with variable ages of onset, such as SMA and lysosomal storage disorders, and parental depression and anxiety is not fully understood. Yet, some have shown that the association does not outweigh the benefits of NBS [31]. It might be inappropriate to apply the general views towards NBS for SMA observed in other countries to the context of Hong Kong. This study aimed to explore the acceptability, ethical considerations, and feasibility of NBS for SMA, especially adult-onset type, in Hong Kong. This is a novel presentation on adult-onset SMA and a comparison between public and healthcare professionals' views.

## Materials and methods

Survey design and population

A prospective cross-sectional online survey was conducted between December 2022 and February 2023 in Hong Kong. The inclusion criteria were the general population aged 18 years or older and healthcare professionals who could be reached by our researchers through email and communication apps. No exclusion criterion was applied. The Qualtrics questionnaire was disseminated via email or hyperlink through social media platforms and communication apps. Responses were collected anonymously, and consent was obtained from participants before they took part in this study.

Questionnaire

The questionnaire was administered using Qualtrics (https://hku.au1.qualtrics.com/jfe/form/SV_8CZzjYykeBNCY2a) and included an information sheet and a two-minute short video (https://drive.google.com/file/d/1SKqFavibXN_8Vm7pz4O3eRk9hWlKtXn8/view) that provided details on clinical symptoms, diagnosis, treatment, and the potential for NBS for SMA. Participants were expected to gain a basic understanding of SMA through either the information sheet or the short video, both of which contained the same information and had been reviewed by chemical pathologists, pediatric and adult neurologists, and public health professionals.

The questionnaire design was adapted from previously published research and was pilot-tested by distributing the first draft to staff at Hong Kong Children’s Hospital, the University of Hong Kong, and the Queen Mary Hospital for feedback [32,33]. Our questionnaire comprised 28 questions. Question 1: consent for participation in the study; questions 2 and 3: whether respondents were SMA patients or their relations with SMA patients; questions 4 and 5: respondent’s knowledge about SMA and its treatment; questions 6-8: respondent’s opinions on supporting NBS for SMA and the reasons behind those opinions. To investigate whether screening for adult-onset SMA is a concern for the general public, respondents were informed that NBS for SMA could detect adult-onset SMA; questions 9-15: from the parents’/patients’ perspective whether positive NBS results should be informed and the reasons behind their choices; question 16: respondent’s preference for timing of treatment if SMA is diagnosed at the pre-symptomatic stage; questions 17-21: retrospectively, whether respondents would want their parents to be informed of their own NBS results as infants, and the reasons behind their choices; and questions 22-28: demographic information. Support or opposition for SMA NBS was assessed using five-point Likert scales and open-ended questions. Descriptive statistics was conducted using Microsoft Excel (Redmond, WA: Microsoft Corp.) to determine the absolute frequencies of responses for each option and percentages for qualitative variables, including responders’ demographic information and perspectives towards the implementation of NBS for SMA.

This study has been approved by the Central Institutional Review Board of Hospital Authority, Hong Kong (#PAED-2022-014). The survey included a consent section and an information sheet that stated the aim of the research and how the interviewee’s response would be used only for research purposes and kept confidential.

## Results

Demographics

A total of 233 valid responses were received, with 82 males (36.8%) and 141 females (63.2%). The majority of respondents were between 18 and 30 years of age (28.3%), Chinese (99.6%), without religious affiliation (52.9%), and had completed tertiary education (42.2%). Among the respondents, 165 were from the general public (74%) and 58 were healthcare professionals (26%). Almost half (47.1%, n=105) indicated that they were parents. Two respondents were SMA patients. About 22% of respondents (n=49) had a relationship with SMA patients, including family members, friends or relatives, legal guardians, patients, healthcare professionals, and others (non-governmental organizations, social workers, and relatives of other rare diseases). Detailed demographic information was summarized in appendix 1.

Knowledge of SMA

A total of 68.6% of the respondents, comprising 99 general public members and 54 healthcare professionals, had some level of knowledge about SMA before participating in this survey. However, almost half of the respondents (49.3%), including 97 general public members and 13 healthcare professionals, were not aware of any new treatments for SMA. A significant majority (83.9%, n=187) believed that SMA should be treated as soon as it is detected through NBS, whereas 16.1% (n=36) thought that treatment should commence only after symptoms appear (Table 1).

**Table 1:** Respondent’s opinions whether they support NBS SMA and timing of treatment (n=223). NBS: newborn screening; SMA: spinal muscular atrophy

Questions	n (%)
If newborn screening is available in Hong Kong, would you have your children screened?	
Yes	211 (94.6)
No	12 (5.4)
If your children were screened positive as SMA patients and the results suggested more likely a later onset at late childhood, adolescence, or adulthood, when would you like him/her to be treated?	
Treat immediately without waiting for symptoms to occur	187 (83.9)
Treat after symptoms occur and diagnosis is made	36 (16.1)

Views of NBS for SMA

An overwhelming majority (94.6%, n=211) of respondents, consisting of 154 general public members and 57 healthcare professionals, supported NBS for SMA (Table 1). Among the reasons for supporting NBS for SMA, 90.5% (n=191) of respondents agreed or strongly agreed with providing immediate treatment for newborns who test positive; 86.2% (n=182) agreed or strongly agreed with screening their children even if the time of symptom onset is uncertain; 79.7% (n=168) agreed or strongly agreed with the non-invasive nature of the screening (Table 2). On the other hand, 5.4% (n=12) of respondents did not support NBS for SMA due to various reasons. Among them, 41.6% (n=5) believed the screening would cause anxiety, 33.4% (n=4) considered the screening unnecessary, 33.3% (n=4) were concerned about the potential discrimination resulting from the screening, and 25% (n=3) believed testing should only be done after symptoms appear (Table 3).

**Table 2:** If you support SMA newborn screening, do you agree the following reasons? (n=211). SMA: spinal muscular atrophy

Variables	Strongly disagree, n (%)	Disagree, n (%)	Neutral, n (%)	Agree, n (%)	Strongly agree, n (%)
My children can be treated immediately if the screening result is positive	10 (4.7)	2 (1.0)	8 (3.8)	37 (17.5)	154 (73.0)
The test is not invasive	11 (5.2)	6 (2.8)	26 (12.3)	48 (22.7)	120 (57.0)
I would like my children to undergo screening even if time of onset of SMA sometimes cannot be exactly determined	9 (4.3)	5 (2.4)	15 (7.1)	52 (25.1)	129 (61.1)

**Table 3:** If you object SMA newborn screening, do you agree the following reasons? (n=12). SMA: spinal muscular atrophy

Variables	Strongly disagree, n (%)	Disagree, n (%)	Neutral, n (%)	Agree, n (%)	Strongly agree, n (%)
This screening is not necessary	1 (8.3)	1 (8.3)	6 (50.0)	2 (16.7)	2 (16.7)
Tests should be done only after symptoms occur	3 (25.0)	1 (8.3)	5 (41.7)	2 (16.7)	1 (8.3)
This screening makes me feel anxious	2 (16.7)	2 (16.7)	3 (25.0)	4 (33.3)	1 (8.3)
Screening result poses a potential for discrimination	2 (16.7)	0 (0)	6 (50.0)	3 (25.0)	1 (8.3)

In the context of parents with children likely to have adult-onset SMA, 87.9% (n=196) of respondents would want to know their child’s NBS result; 78% (n=174) preferred their children to undergo medical surveillance from birth; and 66.8% (n=149) would inform their children of the result. Regarding respondents who considered themselves likely to have adult-onset SMA, 80.7% (n=180) agreed that their parents should be informed of the screening results at the time of the test; and 73.5% (n=164) agreed to undergo medical check-ups and follow-ups from birth (Table 4). The specific reasons for supporting or not supporting NBS for SMA are detailed in appendix 2.

**Table 4:** From the parents’/patients’ perspective whether NBS results should be informed and the reasons behind their choices (n=223). NBS: newborn screening; SMA: spinal muscular atrophy

Questions		n (%)
As parents, if the newborn screening result of your children was very likely to be adult-onset SMA -	Do you want to know the report of your children?	
	Yes	196 (87.9)
	No	15 (6.7)
	I don’t know	12 (5.4)
	Would you tell your children the result?	
	Yes	149 (66.8)
	No	30 (13.5)
	I don’t know	44 (19.7)
	Do you want your children to have medical surveillance since newborn?	
	Yes	174 (78)
	No	22 (9.9)
	I don’t know	27 (12.1)
As yourself, if the newborn screening result of you was very likely to be adult-onset SMA -	Do you want your parents to be informed of the results at the time of screening?	
	Yes	180 (80.7)
	No	18 (8.1)
	I don’t know	25 (11.2)
	Do you want to have a medical checkup and follow-up since newborn?	
	Yes	164 (73.5)
	No	22 (9.9)
	I don’t know	37 (16.6)

## Discussion

This is the first cross-sectional survey study to examine the attitudes of the general public and healthcare professionals in Hong Kong towards NBS for SMA, particularly regarding the detection of adult-onset SMA through NBS, an area that has not received extensive attention in prior research. Although SMA4 is mostly mild, with the majority of patients retaining the ability to walk independently, they may need supportive therapies like physiotherapy and occupational therapy to help maintain their health and improve their quality of life in the latter decades of life. Disease-modifying therapies like nusinersen may also help slow disease progression despite a lack of current evidence regarding their efficacy profile in milder forms of SMA. Other clinical trials are ongoing to identify other potential treatments. Early diagnosis by NBS can aid in treatment planning in this unique group of patients for best medical outcome and therefore we would like to collect opinions on this topic.

This study includes perspectives from both healthcare professionals and the general public, providing a comprehensive view of attitudes toward NBS for SMA. It is crucial to investigate the opinions of both groups since challenges may arise with the current screening method. Consensus between both parties is of utmost importance regarding the successful implementation of the NBS program. Although NBS can detect SMA in patients at a pre-symptomatic stage, it is often challenging to accurately determine the specific type and onset period of SMA. In this study, healthcare professionals showed a greater understanding of SMA, with 47.3% (n=27) possessing extensive knowledge compared to only 16.4% (n=27) of the general public. Nearly all respondents expressed positive views on NBS for SMA, with 94.6% agreeing to have their children screened if NBS were available in Hong Kong.

NBS for various diseases is essential for early monitoring and treatment, with over 94.6% of our respondents supporting this for SMA. A local cross-sectional survey study assessed parental knowledge and attitudes toward an expanded NBS program for inborn errors of metabolism, with 97.7% supporting its implementation [34]. This demonstrates that the majority of Hong Kong people agree with NBS and recognize the importance of early intervention for newborn diseases, which aligns with our survey findings. An Australian non-randomized cohort study on NBS for SMA showed that patients who underwent NBS and early treatment experienced greater improvements in functional burden and associated comorbidities than those without NBS [35]. These improvements include survival rate, motor function, non-invasive ventilation, and feeding support, indicating that NBS for SMA and early treatment are clinically beneficial for patients.

In this survey, we found that most Hong Kong people (94.6%, n=211) supported NBS for all types of SMA (Table 1). Among the supporters, 86.2% (n=181) supported screening even if the time of onset of SMA cannot be exactly determined (Table 2). A UK survey study with 232 respondents unrelated to SMA evaluated their attitudes toward NBS for the condition. Among them, 84% of respondents agreed to NBS, with 82% supporting the importance of identifying SMA at birth, even if the SMA type could not be determined, which also aligns with our survey findings [33]. As our study would like to emphasize the concerns regarding screening of adult-onset SMA, we have 73.5% (n=164) of respondents who would like to have medical checkups and follow-ups if their result was very likely to be adult-onset SMA due to better monitoring and early treatment of the disease (Table 4). Besides, 83.9% (n=187) of our respondents preferred immediate treatment upon a positive screening result including adult-onset SMA (Table 1). The findings indicate a strong local consensus accepting the possibility of picking up adult-onset SMA through NBS for SMA and receiving early treatment to improve the prognosis.

This survey aimed to address ethical concerns regarding informing people of their results, even if they are likely to develop adult-onset SMA. From a parental perspective, they believed this could aid in diagnosing and treating their children and facilitate life planning. Lee et al. reported that over 95% of respondents agreed to NBS and many believed early diagnosis was crucial [32]. This survey also showed that 78% (n=174) of respondents would like to have their children under medical surveillance since birth (Table 4). Medical surveillance can facilitate timely diagnosis when children become symptomatic and provide immediate intervention while reducing time and monetary costs for investigations. Even though a minority in the present study preferred not to be informed of their likely adult-onset SMA status (9.9%, n=22) (Table 4), their main concern was the disruption of normal life (59.1%, n=13) instead of questioning the time of having medical checkup and follow-up (appendix 2). This further demonstrates that early diagnosis of adult-onset SMA is essential to devise medical follow-up plans to safeguard patient’s health. However, we also found that for those who preferred not to be informed, 61.1% (n=11) expressed concern about disruption of normal life and potential discrimination (appendix 2). This is a common concern for any NBS, as seen in fragile X syndrome, where screening can potentially cause stigmatization by disclosing carrier status or adult-onset disorders [36]. To address this, proper education on SMA NBS should be provided to those hesitant about the screening.

We also discussed concerns about the time of treatment. Some recent research supports the proactive approach of early treatment of SMA to reduce symptoms and improve quality of life, especially for patients with four copies of SMN2 genes [28,37], despite a higher likelihood of SMA3 than SMA2 in patients with four copies of SMN2 genes [38]. Table 5 summarizes the approach of different countries to give treatment regarding the copy number of SMN2 gene or type of onset. At this stage, treatments are indicated in all types of SMA in the United States and Japan, while treatments are also indicated in Europe for SMA patients carrying up to four copies of SMN2 genes. As this study showed that most people supported NBS for SMA including the possibility of detecting adult-onset type as well as early diagnosis and treatment, treatment should consider at least giving SMA patients with four copies of SMN2 in Hong Kong to have a better prognosis. Although there is no data currently available concerning treatment efficacy in SMA 4, it is believed that nusinersen may be most effective when started as soon as possible after diagnosis.

**Table 5:** Initiation of treatment of SMA based on SMA type or SMN2 copies. *According to the European Medicines Agency. **Gene therapy, other than onasemnogene abeparvovec, is used for pre-symptomatic and early symptomatic SMA patients less than one year of age, diagnosed by NBS, with SMN2 copy 1-3. The symbol "-" refers to information that is not indicated.

Countries	Nusinersen		Onasemnogene abeparvovec		Risdiplam	
	Type	SMN2 copies	Type	SMN2 copies	Type	SMN2 copies
Australia [39]	I-III	1-2	I	1-3	I-III	-
Europe*	I-III	1-4	I	1-2	I-III	1-4
Japan [40]	I-IV	-	I	-	I-III	-
United Kingdom [41]	I-III	-	I-III	-	I-III	-
United States	I-IV	-	I-IV	-	I-IV	-
China [29]	I-III	1-4	N/A**	N/A	I-III	1-4

One concern regarding immediate treatment is the financial burden it places on patients and their families. In the present survey, almost half of the 22 people who wished not to have their child to receive medical surveillance since birth for adult-onset SMA were concerned about the financial burden. The high cost of the three currently available drugs is a significant factor in this concern. However, in China, the government heavily reimburses the cost of nusinersen, reducing the price per dose significantly for the patients [42]. This suggests that more research is needed on the feasibility and cost-benefit analysis of current reimbursement policies to guide the potential ways to relieve the financial burden of the families of SMA.

Regarding the association between anxiety and NBS, it is observed that for respondents who objected NBS for SMA (5.4%, n=12), five of them felt anxious about the screening, while seven of them were either neutral or not anxious about the screening (Table 3). For parents who did not wish to know the report of their children (6.7%, n=15), only seven of them felt anxious about their children’s results while eight of them felt either neutral or not anxious. However, 21 out of 30 respondents who would not tell their children the screening result tended to think that the newborn screening would make their children anxious (appendix 2). Due to the majority of subjects supporting NBS for SMA, the above figures cannot show the association between anxiety and NBS.

Limitations

There are several limitations to our survey. Firstly, the sample size was relatively small, which may affect the generalizability of the findings. Secondly, the distribution of participants was uneven, with nearly 75% from the general public. This imbalance between healthcare professionals and general public may raise questions about the potential influence of educational background and knowledge on preferences for NBS. However, the present study demonstrated that most people would support NBS for SMA, regardless of whether they were healthcare professionals or the general public. Thirdly, the geographical limitation to Hong Kong may restrict the applicability of our findings in other contexts. While the insights gained are valuable, they may not be able to represent attitudes in different cultural or healthcare environments. Future research by conducting larger-scale studies may enhance the generalizability of the findings. More in-depth qualitative analyses in future studies may also provide richer insights into the motivations and concerns of different demographic groups regarding NBS for SMA. Besides, this study may contain potential biases as it is a cross-sectional study. To address this, we referred to similar studies to develop our questionnaire. Finally, the system does not have measures to exclude the possibility of multiple responses from a single individual.

## Conclusions

In conclusion, this study provides new insights into the local population’s perspectives regarding NBS of SMA, with novel focus on adult-onset SMA. We found that healthcare professionals showed a greater understanding of SMA compared to the general public due to their expert knowledge. Despite this, the majority of people support the implementation of NBS for SMA in Hong Kong regardless of their demographic backgrounds. Most participants demonstrated a positive mindset, believing that early diagnosis and treatment planning can maintain health and improve the quality of life in SMA patients, especially in adult-onset type who may potentially live a normal life with early intervention. However, the limited sample size in this study necessitates cautious interpretation of such apparent general acceptance of NBS for adult-onset SMA. Concerns about not supporting NBS for SMA, such as anxiety regarding screening results and the timing of treatments, can be addressed through public education. The current findings are specific to the context of Hong Kong and may not be applicable elsewhere. Future research by conducting larger-scale studies with in-depth qualitative analyses may enhance the generalizability of the survey findings and provide richer insights into the motivations and concerns of different demographic groups regarding NBS for SMA.

## Appendices

Appendix 1

**Table 6:** Respondents demographics (n=223). SMA: spinal muscular atrophy

Variables	n (%)
Sex	
Male	82 (36.8)
Female	141 (63.2)
Age (years)	
18-30	63 (28.3)
31-40	50 (22.4)
41-50	61 (27.4)
51-64	38 (17.0)
65 or above	11 (4.9)
Do you have children?	
Yes	105 (47.1)
No	118 (52.9)
Ethnicity	
Chinese	222 (99.6)
Non-Chinese	1 (0.4)
Religious affiliation	
No religious preference	118 (52.9)
Christian	79 (35.4)
Catholic	10 (4.5)
Buddhist	12 (5.4)
Taoist	2 (0.9)
Hindu	0 (0)
Muslim	2 (0.9)
Jewish	0 (0)
Others	0 (0)
Highest level of education	
Primary school	0 (0)
Secondary school	50 (22.4)
Post-secondary (diploma/associate degree)	27 (12.1)
Tertiary/Bachelor (University)	94 (42.2)
Master’s degree or above	52 (23.3)
Occupation	
Healthcare provider	58 (26.0)
Others	165 (74.0)
Are you a SMA patient?	
Yes	2 (0.9)
No	221 (99.1)
Do you have any relationship with SMA patients?	
Family members	8 (3.6)
Friends or relatives	9 (4.0)
Legal guardian	1 (0.4)
Patient and healthcare professional	26 (11.7)
No relationship	174 (78.0)
Others	5 (2.3)

Appendix 2

**Table 7:** Specific reasons for supporting or not supporting NBS for SMA and respondents attitudes towards disclosure of positive NBS results. NBS: newborn screening; SMA: spinal muscular atrophy

Variables		Strongly disagree, n (%)	Disagree, n (%)	Neutral, n (%)	Agree, n (%)	Strongly agree, n (%)
If you want to know the report of your children, do you agree on the following reasons? (n=196)	Diagnosis can be made quickly when symptoms occur	6 (3.1)	1 (0.5)	6 (3.1)	35 (17.8)	148 (75.5)
	Treatments can be started as soon as symptoms occur	6 (3.1)	1 (0.5)	4 (2.0)	33 (16.9)	152 (77.5)
	It can lead to better support for children	6 (3.1)	1 (0.5)	6 (3.1)	32 (16.3)	151 (77)
	It enables parents to make informed decisions about future pregnancy	6 (3.1)	2 (1.0)	13 (6.6)	39 (19.9)	136 (69.4)
If you do not want to know the report of your children, do you agree on the following reasons? (n=15)	This screening is not necessary	4 (26.7)	0 (0)	5 (33.3)	6 (40.0)	0 (0)
	This screening makes me feel anxious	2 (13.3)	0 (0)	6 (40.0)	3 (20.0)	4 (26.7)
	Screening results of adult-onset SMA should not be included	3 (20.0)	1 (6.7)	5 (33.3)	6 (40.0)	0 (0)
	Screening results can increase my insurance premium and narrow my insurance coverage	2 (13.4)	0 (0)	5 (33.3)	5 (33.3)	3 (20.0)
If you would tell your children the result, do you agree on the following reasons? (n=149)	Children should know their own health condition	4 (2.7)	1 (0.7)	3 (2.0)	29 (19.4)	112 (75.2)
	Children can be better mentally prepared	4 (2.7)	1 (0.7)	4 (2.7)	26 (17.4)	114 (76.5)
	This can make children’s life planning easier	4 (2.7)	2 (1.4)	4 (2.7)	29 (19.4)	110 (73.8)
If you would not tell your children the result, do you agree on the following reasons? (n=30)	This screening makes children feel anxious	2 (6.7)	2 (6.7)	5 (16.6)	6 (20)	15 (50)
	Screening results can make children’s life planning difficult	3 (10.0)	3 (10.0)	4 (13.3)	11 (36.7)	9 (30.0)
	Screening results can disrupt children’s normal life	3 (10.0)	4 (13.3)	5 (16.7)	9 (30.0)	9 (30.0)
	Screening results pose a potential for discrimination	7 (23.3)	1 (3.33)	8 (26.7)	5 (16.7)	9 (30.0)
If you want your children to have medical surveillance since newborn, do you agree on the following reasons? (n=174)	This can better monitor children’s disease condition	6 (3.4)	1 (0.6)	6 (3.4)	22 (12.6)	139 (80)
	Children can have early treatment	6 (3.4)	1 (0.6)	5 (2.9)	17 (9.8)	145 (83.3)
	This reduces children’s suffering when symptoms of SMA occur	6 (3.4)	1 (0.6)	7 (4.0)	20 (11.5)	140 (80.5)
	Regular medical checkups and follow-ups allow early detection of symptoms/signs and timely treatment	6 (3.4)	1 (0.6)	2 (1.1)	25 (14.4)	140 (80.5)
If you do not want your children to have medical surveillance since newborn, do you agree on the following reasons? (n=22)	Early medical checkups and follow-ups cannot help	3 (13.7)	4 (18.2)	8 (36.4)	6 (27.2)	1 (4.5)
	Medical checkups and follow-ups should be done only after symptoms occur	3 (13.7)	7 (31.8)	5 (22.7)	7 (31.8)	0 (0)
	This can pose a financial burden on me	2 (9.1)	4 (18.2)	6 (27.2)	8 (36.4)	2 (9.1)
	Early medical checkups and follow-ups interfere with the normal life of children	1 (4.5)	4 (18.2)	4 (18.2)	8 (36.4)	5 (22.7)
If you want your parents to be informed of the results at the time of screening, do you agree on the following reasons? (n=180)	Diagnosis can be made quickly when symptoms occur	7 (3.9)	2 (1.1)	5 (2.8)	20 (11.1)	146 (81.1)
	Treatments can be started as soon as symptoms occur	7 (3.9)	1 (0.5)	7 (3.9)	25 (13.9)	140 (77.8)
	It enables me to make informed decisions about future pregnancy	6 (3.3)	2 (1.1)	12 (6.7)	23 (12.8)	137 (76.1)
If you do not want your parents to be informed of the results at the time of screening, do you agree on the following reasons? (n=18)	Screening results can disrupt my normal life	0 (0)	1 (5.6)	4 (22.2)	6 (33.3)	7 (38.9)
	Screening results can make my life planning difficult	0 (0)	0 (0)	5 (27.8)	6 (33.3)	7 (38.9)
	Screening results pose a potential for discrimination	2 (11.1)	1 (5.6)	4 (22.2)	2 (11.1)	9 (50.0)
	Screening results can increase my insurance premium and narrow my insurance coverage	0 (0)	2 (11.1)	3 (16.7)	4 (22.2)	9 (50.0)
If you want to have medical checkups and follow-ups since newborn, do you agree on the following reasons? (n=164)	This can better monitor disease condition	5 (3.0)	0 (0)	7 (4.3)	22 (13.4)	130 (79.3)
	I can have early treatment	5 (3.0)	0 (0)	5 (3.0)	21 (12.8)	133 (81.2)
	This reduces my suffering when symptoms of SMA occur	5 (3.0)	0 (0)	6 (3.7)	18 (11.0)	135 (82.3)
	Regular medical checkups and follow-ups allow early detection of symptoms/signs and timely treatment	5 (3.0)	0 (0)	5 (3.0)	17 (10.4)	137 (83.6)
If you do not want to have medical checkups and follow-ups since newborn, do you agree on the following reasons? (n=22)	Early medical checkups and follow-ups cannot help	3 (13.6)	5 (22.8)	7 (31.8)	4 (18.2)	3 (13.6)
	Medical checkups and follow-ups should be done only after symptoms occur	3 (13.6)	4 (18.2)	7 (31.8)	6 (27.3)	2 (9.1)
	This can pose a financial burden for me	5 (22.8)	1 (4.5)	7 (31.8)	7 (31.8)	2 (9.1)
